# Enumerating Finitary Processes

**DOI:** 10.3390/e26121105

**Published:** 2024-12-17

**Authors:** Benjamin D. Johnson, James P. Crutchfield, Christopher J. Ellison, Carl S. McTague

**Affiliations:** 1 Complexity Sciences Center, University of California at Davis, One Shields Avenue, Davis, CA 95616, USA; 2 Mathematics Department, University of California at Davis, One Shields Avenue, Davis, CA 95616, USA; 3 Physics Department, University of California at Davis, One Shields Avenue, Davis, CA 95616, USA; 4 DPMMS, Centre for Mathematical Sciences, University of Cambridge, Wilberforce Road, Cambridge CB3 0WB, UK

**Keywords:** epsilon-machine, orderly enumeration, 02.50.-r, 89.70.+c, 05.45.Tp, 02.50.Ey

## Abstract

We show how to efficiently enumerate a class of finite-memory stochastic processes using the causal representation of ϵ-machines. We characterize ϵ-machines in the language of automata theory and adapt a recent algorithm for generating accessible deterministic finite automata, pruning this over-large class down to that of ϵ-machines. As an application, we exactly enumerate topological ϵ-machines up to eight states and six-letter alphabets.

## 1. Introduction

What does the landscape of stochastic processes look like? Some classes of process—e.g., modeled by Markov and Hidden Markov chains, finite or denumerable [1,2,3,4]—are familiar to us since they have proven so useful as models of randomness in real world systems. Even if this familiarity belies a now-extensive understanding for particular classes, it begs the question of the intrinsic organization and diversity found in the space of all stochastic processes. Randomly selecting a stochastic process, how often does one find that it saturates the entropy rate? How many distinct processes are there at a given entropy rate or with a given number of states? Answers to these and related questions will go some distance to understanding the richness of stochastic processes and these, in turn, will provide hints as to what is possible in nature.

Stochastic processes show up in an exceedingly wide range of fields, but they are not generally analyzed or classified in broad swaths. In an attempt to address such concerns, we show how to enumerate the class of stochastic processes that admit the causal representation of finite-state ϵ-machines.

An ϵ-machine is the minimally complex, maximally predictive representation that completely captures all of a stochastic process’s information storage and processing properties. The ϵ-machine representation allows for the direct analysis of the underlying process using only relevant information, and it provides a framework for comparing different processes through common, measurable quantities, such as process entropy rate and statistical complexity. The literature on computational mechanics [5], the area responsible for the theory of ϵ-machines, provides details about the construction of ϵ-machines from process output, proof of their optimality, various information-theoretic quantities that can be calculated from the ϵ-machine, and more.

Here, we consider stationary stochastic processes over discrete states, alphabets, and time. Given that each such process can be completely represented by its ϵ-machine, to enumerate all stochastic processes it suffices to enumerate all ϵ-machines. Even if one restricts to the case of ϵ-machines with finitely many states, this task appears to be extraordinarily difficult. So, as a first step, we enumerate a subclass of ϵ-machines called topological ϵ-machines, which represent a subclass of all finite-memory processes. In a sequel, we extend the ideas presented here to more general stochastic processes and their ϵ-machines.

Although we are a long way from mapping the landscape of all stochastic processes, enumerating a subclass of finite-memory stochastic processes is useful for a number of reasons. The first is basic understanding. One would simply like to know how many processes there are for a given number of states and alphabet size. Moreover, if we fix one of these parameters and increase the other, it is informative to see how the number of distinct processes scales as well. Second, it allows for a thorough survey of process characteristics. An example of a such a survey is found in Ref. [6]. Third, an enumerated list of processes can be used to rigorously establish properties for various kinds of complex systems. For example, proving theorems about pattern formation in cellular automata. Finally, and rather more generally, one needs to be able to sample and explore the space of processes in a random or a systematic way, such as required in Bayesian inference.

Starting from an algorithm initially designed to enumerate deterministic finite automata, we use ϵ-machine properties as selection criteria for these automata, resulting in the set of topological ϵ-machines (and the processes they describe) as a result. Our development of this is organized as follows. First, we briefly discuss our previous approach to this problem using a different orderly enumeration algorithm due to Read [7], followed by an overview of the algorithm on which our enumeration scheme is based [8,9]. Second, we lay out the machinery of this algorithm, reviewing automata theory and computational mechanics. We define the necessary concepts as they apply to ϵ-machine generation and enumeration. Third, we then describe our algorithm, give the pseudocode for its implementation, and prove that it successfully enumerates all topological ϵ-machines. Fourth, we present enumeration results as a function of the number of states and symbols. We discuss, as well, the performance of the new algorithm, comparing it to our previous algorithm, and explain the improvements.

## 2. Related Work

The enumeration of ϵ-machines has not, to our knowledge, been previously explored, outside of the above-cited works. The enumeration of certain classes of automata, in contrast, has been pursued with varying degrees of success. Of particular interest, strongly connected and minimal complete finite automata were separately enumerated in Refs. [10,11], respectively. See Ref. [12] and references therein for more details on other recent efforts.

Much of the literature on computational mechanics focuses on ϵ-machines from the standpoint of Markov chains and stochastic processes and, therefore, typically uses the transition matrices as an ϵ-machine’s representation. Our first approach for enumerating finitary processes focused on generating all possible transition matrices and, hence, all ϵ-machines, interpreted as labeled directed graphs. Read [7] presented an orderly generation algorithm that could be used to efficiently generate certain classes of combinatorial objects. Among the objects that can be generated are directed and undirected graphs, rooted trees, and tournaments (interpreted as a special class of directed complete graphs). The essence of Read’s algorithm is that, given the complete list $L_{m}$ of graphs with *n* nodes and *m* edges, we can construct the complete list $L_{m+1}$ of graphs with *n* nodes and m+1 edges without having to run an isomorphism check against each of the already constructed graphs. This offers a significant speed improvement versus the classical method.

We initially adapted Read’s algorithm to generate all edge-labeled multi-digraphs (with loops). From this extensive list, we then eliminated graphs that were not strongly connected and minimal in the sense of finite automata theory. While this algorithm was successful, it had three main performance drawbacks: (1) a large memory footprint, as $L_{m}$ must be stored to generate $L_{m+1}$; (2) an improved but still extensive isomorphism check for each generated graph—the worst-case scenario requires n! comparisons for each generated graph; and (3) the generation of a substantially larger class than needed and, as a consequence, many graphs to eliminate.

Our second approach, and the one presented in detail here, uses a different representation of ϵ-machines, looking at them as a type of deterministic finite automata (DFA). The new algorithm suffers from none of the previous method’s problems, although it should be noted that this method cannot be used to enumerate the generalized structures available via Read’s algorithm.

In his thesis, Nicaud [13] discussed the enumeration of “accessible” DFAs restricted to binary alphabets. These results were then independently extended to *k*-ary alphabets in Refs. [14,15]. Recently, Almeida et al. [8,9] developed an algorithm that generates all possible accessible DFAs with *n* states and *k* symbols using a compact string representation initially discussed in Refs. [8,9]. They showed that considering the “skeleton” of these DFAs as *k*-ary trees with *n* internal node guarantees that a DFA’s states are all accessible from a start state. From there, they procedurally add edges to the tree in all possible ways to generate all DFAs. As it is possible to generate all such trees, they show that it is possible to generate all accessible DFAs. They continue on to discuss their enumeration in comparison to the methods of Refs. [14,15], as well as giving a brief commentary on the percentage of DFAs that are minimal for a given number of states and symbols.

## 3. Automata Representations

We start with notation and several definitions from automata theory [16] that serve as the basis for the algorithm.

**Definition 1.**  *A deterministic finite automaton is a tuple $\langle Q,\Sigma ,\delta ,q_{0},F\rangle$, where Q is a finite set of states,* Σ *is a discrete alphabet, δ:Q×Σ→Q is the transition function, $q_{0}$ is the start state, and F⊆Q is the set of final (or accepting) states. (Technically speaking, despite being referred to as a transition function, δ(·) is a partial function—some outputs are not specified).*

Informally, we extend the transition function from individual symbols to words with δ(q,λ)=q, for all q∈Q, and for v,v′∈Σ,δ(q,vv′)=δ(δ(q,v),v′). Here, λ denotes the empty word. (Note that the transition partial function on symbols can be made a total function on words [8,9]).

With |Q|=n and |Σ|=k, we take our set of states to be Q={0,…,n−1} and our alphabet to be Σ={0,…,k−1}. When context alone is not clear, states and symbols will be denoted by $q_{i}$ and $v_{j}$, respectively. We will use F=Q (all states are accepting) for our algorithm, although this is not a general characteristic of DFA but is a property of ϵ-machines.

**Definition 2.**  *A DFA is complete if its transition function δ is total. That is, for any state q∈Q and symbol v∈Σ,δ(q,v)=q′ for some q′∈Q.*

The DFAs generated by Refs. [8,9]’s algorithm may be incomplete. Shortly, we will see this is a necessary condition for the DFA to be a topological ϵ-machine.

**Definition 3.**  Two states, q and q′, of a DFA are said to be equivalent if for all words $w\in \Sigma^{*}$, δ(q,w)∈F if and only if δ(q′,w)∈F. That is, for every word w, following the transitions from q and q′ both lead to accepting or nonaccepting states. A DFA is minimal if there are no pairwise equivalent states.

As we take F=Q for ϵ-machines, we can simplify the idea of equivalence somewhat.

**Definition 4.**  *Two states of a topological ϵ-machine are equivalent if the sets of words following each state are the same.*

**Definition 5.**  A DFA is accessible or initially connected if for any state q∈Q, there exists a word $w\in \Sigma^{*}$ such that $\delta (q_{0},w)=q$.

Simply put, there is a directed path from the initial state to any other state. The reverse is not necessarily true.

**Definition 6.**  A DFA is strongly connected if for any two states q,q′∈Q, there is a word $w\in \Sigma^{*}$ such that δ(q,w)=q′. Equivalently, for any state q∈Q, setting $q_{0}=q$ results in the DFA still being accessible.

**Definition 7.**  *Two DFAs are isomorphic if there is a one-to-one map between their state sets that (i) maps accepting states of one DFA to the corresponding states of the other, (ii) similarly with their nonaccepting states, (iii) preserves adjacency, and (iv) preserves edge labeling when applied to δ.*

**Definition 8.**  *A finite ϵ-machineis a probabilistic finite-state machine with a set of n causal states $\left\{\sigma_{1},\ldots ,\sigma_{n}\right\}$, a finite alphabet of k symbols $\left\{v_{1},\ldots ,v_{k}\right\}$, and a set of k n×n symbol-labeled substochastic transition matrices $T^{\left(v_{i}\right)}$, i∈{1,…,k}. Here, $T_{\sigma ,\sigma^{\prime }}^{\left(v\right)}$ is the probability of transitioning from state σ to state $\sigma^{\prime }$ while emitting symbol v.*

*Moreover, there are two key additional defining properties. The first is unifilarity: for each state and each symbol, there is at most one outgoing transition. Second is probabilistically distinct states: for each pair of distinct states σ and $\sigma^{\prime }$, there exists a finite word $w=v_{1}\ldots v_{\ell }$ such that $\mathrm{Pr}\left(w|\sigma \right)\neq Pr\left(w|\sigma^{\prime }\right)$.*

Due to these properties, one can show that the ϵ-machine is minimal [5].

An ϵ-machine has transient and recurrent components, but we only focus on the recurrent portion, as the transient component can be calculated from the recurrent. In the following, when we talk about ϵ-machines, we implicitly refer to the recurrent states. With this restriction, ϵ-machines are also strongly connected.

**Figure 1:**
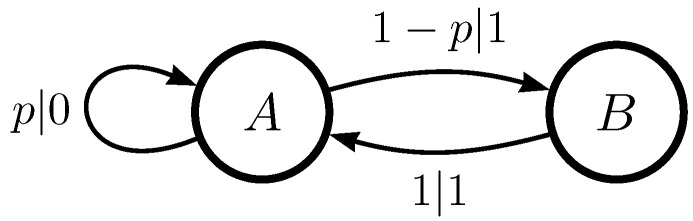
Even Process. The transition labels p|x denote the probability p∈(0,1) of generating symbol *x*.

Figure 1 gives the ϵ-machine for the *Even Process*. The Even Process produces binary sequences in which all blocks of uninterrupted 1 s are even in length, bounded by 0 s. Furthermore, after each even length is reached, there is a probability *p* of breaking the block of 1 s by inserting a 0. If a 0 is inserted, then the same rule applies again.

**Definition 9.**  *A topological ϵ-machine is an ϵ-machine where the transition probabilities from a single state are uniform across all outgoing edges.*

**Figure 2:**
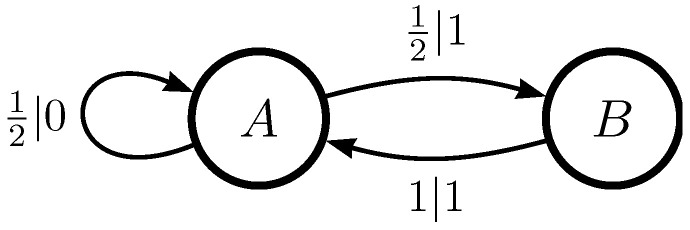
Topological ϵ-machine for the Even Process. Transition probabilities are uniform across edges leaving state *A*.

The topological ϵ-machine for the Even Process is given in Figure 2. We see that the transitions on both edges leaving state *A* have probability 1/2, instead of *p* and 1−p as they were in the original Even Process ϵ-machine.

Since the transition probabilities are uniform across all edges leaving each single state, we only need to know their number. As far as the enumeration algorithm is concerned, we may effectively ignore the probabilities and focus instead on where the edges go.

This makes clear the name topological ϵ-machine: we are only interested in the topological structure (connectivity or adjacency) as this determines all its other properties.

One of the key reasons for the success of the algorithm is its compact representation of DFAs, which allows for direct enumeration. Recall that |Σ|=k and suppose that there is a fixed ordering 0,…,n−1 on the states *Q*.

**Definition 10.**  *A DFA’s string $S=[t_{0},t_{1},\ldots ,t_{nk-1}]$ is an nk-tuple that specifies the terminal state $t_{i}\in Q$ on each outgoing edge. The first k entries in the string correspond to the states reached by following the edges labeled 0,…,k−1 that start in state 0. The next k t_i_s correspond to the edges that start in state* 1 *and so on. Thus, for each of the n states, there are k specified transitions. If an outgoing edge does not exist, the corresponding index is marked with $t_{i}=-1$.*

For clarity, let us consider the topological ϵ-machine for the Even Process. Let states *A* and *B* be denoted by 0 and 1, respectively. The transition symbols will also be 0 and 1, though there is no connection between the two labelings. As *A* transitions to *A* on a 0 and to *B* on a 1, the terminal states for these two transitions are 0 and 1, respectively. *B* has no outgoing transition on symbol 0, so that will be denoted −1 in the string, while the transition from *B* to *A* on a 1 will be given by 0. Thus, the string representation for the Even Process is S=[0,1,−1,0].

In the definition of a DFA’s string, we assumed a fixed ordering on the states. In general, there are n! ways to label the states and as many strings, so we need a way to fix a labeling unambiguously. To do this, we label the states in the order in which they are reached by following edges lexicographically from state $q_{0}$. Start with $q_{0}\equiv 0$, then follow the edges coming out of $q_{0}$ in order: 0,1,…,k−1. The first state reached that is not state 0 is labeled as 1. The next state that is not 0 or 1 becomes state 2, and so on. Once the edges 0,…,k−1 have been explored, the procedure is repeated, starting from state 1, then state 2, and so on, until all the states have been labeled. Given the initial state $q_{0}$ of an accessible DFA, the edges uniquely determine the labeling of all the other states in the DFA. A proof can be found in Refs. [8,9]. Note that the DFA must be accessible for this to work, or else states will be missed in the labeling process.

**Definition 11.**  *Given a DFA string S, the corresponding flag $f=[f_{0},f_{1},\ldots ,f_{n}]$ is an n+1 tuple, with $f_{0}=-1$, $f_{n}=nk$, and $f_{i}=\min\left\{j:S_{j}=i\right\}$. That is, $f_{i}$ is the index of the first occurrence of i in the DFA string S. Note that as the DFA is accessible, $f_{i}\leq ik-1$.*

The flag for the Even Process shown above is [−1,1,4].

## 4. Enumeration Algorithm

To enumerate and generate all topological ϵ-machines, we begin with the Almeida et al. algorithm [8,9] that generates all accessible DFAs, of which topological ϵ-machines are a subclass. We then eliminate those DFAs that are not ϵ-machines. The following Lemmas help with this process.

**Lemma 1.**  *A topological ϵ-machine with n states has at least n transitions.*

**Proof.**  Assume there are at most n−1 transitions. Then, there is at least one state with no outgoing transition. There is no path from this state to any other state, so this cannot be an ϵ-machineas it is not strongly connected.    □

**Lemma 2.**  *A topological ϵ-machine with n>1 states and alphabet size k can have at most nk−1 transitions.*

**Proof.**  The number of transitions is at most nk, as each state can have at most *k* transitions. Suppose that an ϵ-machine has nk transitions. Then, every word $w\in \Sigma^{*}$ is accepting for every state, so all states are pairwise equivalent. This cannot be an ϵ-machine, since it is not minimal. Thus, there are at most nk−1 transitions.    □

This establishes our earlier claim that topological ϵ-machines are incomplete.

**Lemma 3.**  *A topological ϵ-machine with n states has n isomorphic string automata representations.*

**Proof.**  An ϵ-machine is strongly connected. In the above definition of a strongly connected DFA, we gave an equivalent characterization where any state may serve as $q_{0}$ and result in an accessible DFA. As state $q_{0}$ determines the labeling of the states, and so the string representations, there are exactly *n* such representations.    □

We now need to determine the canonical representation for a given topological ϵ-machine. Given the *n* different strings that all represent the ϵ-machine equally well, which do we add to our enumerated list, and how do we know if we already have an isomorphism of an ϵ-machine on our list?

A closed-form expression to exactly count the number $B_{n,k}^{1}$ of incomplete, accessible DFAs with *n* states and alphabet size *k* was developed in Refs. [8,9]. A bijection between the integers $0,\ldots ,B_{n,k}^{1}-1$ and the DFAs generated by the algorithm was also given. In this way, we can determine the *i*th DFA generated by the algorithm and likewise, given an arbitrary accessible DFA, we can determine exactly where in the generation sequence it occurs. This bijection allows us to easily determine whether an ϵ-machine is the canonical representation for its isomorphism class. We denote by $B_{n,k}^{1}\left(S\right)$ the index of the string representation *S* in the enumeration process. Appendix A gives the details.

**Definition 12.**  *Given the n different string representations of a topological ϵ-machine—$S_{1},S_{2},\ldots$, $S_{n}$—the canonical representation $\hat{S}$ is the string with the smallest $B_{n,k}^{1}$ value. It is the first of the isomorphisms generated by the enumeration process:*

$$\begin{array}{c} \hat{S}\equiv \min_{1\leq i\leq n}B_{n,k}^{1}\left(S_{i}\right)\;. \end{array}$$

With this definition of a canonical representation, it is simple to determine whether a given ϵ-machine has already been generated: compute the index $B_{n,k}^{1}\left(S\right)$ of its representation *S*. Take each state as $q_{0}$ and compute the new string representation. If any of the resulting representations has a lower index than the original, then the given ϵ-machine is not canonical. So, we ignore it and generate the next DFA in the enumeration sequence.

**Figure 3:**
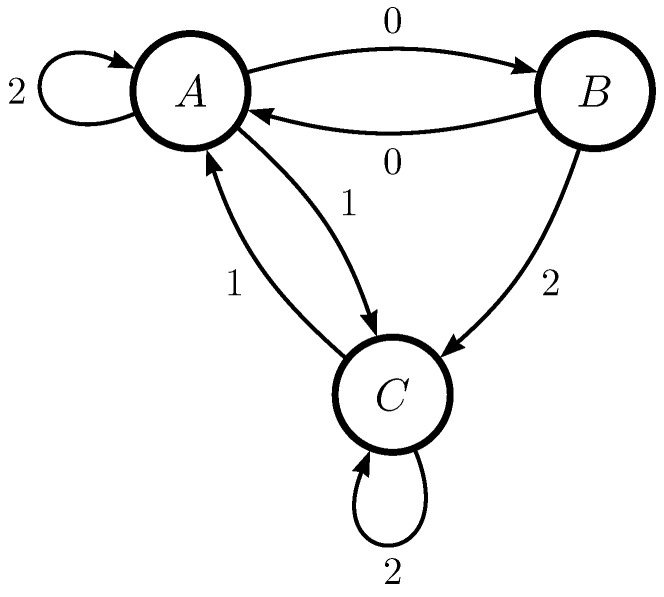
Arbitrary topological ϵ-machine with three states over alphabet of size three.

To solidify the above ideas, consider the topological ϵ-machine in Figure 3. Note that since transition probabilities are not relevant to the enumeration process, we omit them entirely and only show the output symbol. Also, note that we label our states with letters, not numbers, for clarity.

Depending on the choice of $q_{0}$, there are three different representations of this ϵ-machine:

1. $q_{0}=A$:
  To determine the state ordering, we follow the edge labeled 0 and obtain $q_{1}=B$. We follow the edge labeled 2 from state *B* to obtain $q_{2}=C$. In this way, we identify (A,B,C) as (0,1,2) and obtain the string representation $S_{1}=\left[1,2,0,0,-1,2,-1,0,2\right]$. From this, we compute that $B_{n,k}^{1}\left(S_{1}\right)=70791$.
2. $q_{0}=B$:
  We find that $q_{1}=A$ and $q_{2}=C$. So, we identify (A,B,C) as (1,0,2) and determine that $S_{2}=\left[1,-1,2,0,2,1,-1,1,2\right]$. This yields $B_{n,k}^{1}\left(S_{2}\right)=55115$.
3. $q_{0}=C$:
  We identify (A,B,C)=(1,2,0), finding that $S_{3}=\left[-1,1,0,2,0,1,1,-1,0\right]$ and $B_{n,k}^{1}\left(S_{3}\right)=18977$.

All three strings are valid representations of the ϵ-machine, but the third $S_{3}$ has the lowest index (18,977) in the enumeration sequence, so it is the canonical representation of the ϵ-machine. During the enumeration process, the other two representations would be ignored after it was determined they were noncanonical.

With this information in-hand, we can now provide the pseudocode for our algorithm. For clarity of discussion, we break the algorithm into two pieces (Algorithms 1 and 2). The first generates accessible DFAs, while the second tests to see if they are topological ϵ-machines.
**Algorithm 1** DFA Generation**Input**: Number of states *n*, alphabet size *k*.1.Generate the flags in reverse lexicographic order.2.For each flag:
(a)Generate strings with this flag one at a time, in lexicographic order. Each is generated from the previous.(b)Test the DFA string *S* to see if it is a canonical topological ϵ-machine. (See Algorithm 2).(c)If the DFA is canonical, output $B_{n,k}^{1}\left(S\right)$ to the list of topological ϵ-machines.(d)Move to the next flag when all of the strings have been generated.
3.Terminate after the last string when the last flag has been generated.**Output**: The list of indices $\left\{B_{n,k}^{1}\left(S\right)\right\}$ of all topological ϵ-machines for the given *n* and *k*.

**Algorithm 2** Test for topological ϵ-machine
**Input**: DFA *X* in string representation *S* and $B_{n,k}^{1}\left(S\right)$.
1.Reject *X* unless it has at least *n* transitions.2.Reject *X* if it has nk transitions.3.For i=1,…,n−1:(a)Create a new DFA $Y_{i}$ from DFA *X* with $q_{0}=i$.(b)Reject *X* if the states of $Y_{i}$ cannot be labeled by follow edges lexicographically from $q_{0}$.(*X is not strongly connected*).(c)Build string $S_{i}$ for $Y_{i}$.(d)Compute index $B_{n,k}^{1}\left(S_{i}\right)$.(e)Reject *X* if $B_{n,k}^{1}\left(S_{i}\right)\leq B_{n,k}^{1}\left(S\right)$.(*X is not canonical*).
4.Reject *X* if it is not a minimal DFA, based on failing Nerode equivalence [16].**Output**: True or False, whether the input DFA is a canonical representation of a topological ϵ-machine.


We only highlight the important aspects of the DFA generation algorithm here. For a more complete discussion, as well as code for implementation, see Refs. [8,9].

Note that steps 1 and 2 are not formally necessary for the algorithm to work, as any DFA that fails these will be not strongly connected and nonminimal, respectively. However, it is quicker to perform these tests than it is to check for connectedness or minimality, and it is for these reasons that Lemmas 1 and 2 were mentioned.

**Proposition 1.**  *The above algorithm generates all topological ϵ-machines with n states and k symbols.*

**Proof.**  It was already shown in Refs. [8,9] that the original algorithm generates all accessible DFAs with *n* states and *k* symbols. We need only show that our additions result in only topological ϵ-machines being generated.

As stated previously, topological ϵ-machines are minimal and strongly connected. We also require a single representative of an isomorphism class. We check that we only obtain strongly connected DFAs in step 3(b), and we obtain minimality from step 4. Finally, we prune state isomorphisms with the test in step 3(e). □

See Ref. [16] for details on the minimization algorithm used here. Also, note that we are not interested in the minimal DFA itself, only whether the given DFA *is* minimal. We minimize the automaton and accept it if it has the same number of states as the original.

Note that the order of the above checks for connectedness, minimality, and isomorphic redundancy can be changed, but the performance of the algorithm suffers. The minimization algorithm is the slowest step, so it should be performed as few times as necessary, which is why it appears last.

## 5. Results

We ran the algorithm on a range of *n* and *k* values. To date, the majority of work in computational mechanics has focused on binary alphabets, so we provide not only the number $E_{n,2}$ of ϵ-machines with a binary alphabet but also a breakdown by the number of edges (transitions) for a given number of states in Table 1. 

Looking at the numbers in the table, we see that the number of ϵ-machines increases quite rapidly, but when compared to the total number $B_{n,2}^{1}$ of accessible binary DFAs, the ratios decrease. At n=3, 9.6% of all accessible DFAs were topological ϵ-machines, while at n=8, that ratio was already down to 3.8%. We also see that for any given number of states, the majority of ϵ-machines have the maximum number of possible edges. This is not surprising as a DFA is more likely to be strongly connected with more edges present.

We note that $E_{n,2}$ is now listed on the *On-Line Encyclopedia of Integer Sequences* as sequence A181554 [17].

We can certainly consider larger alphabets, and Table 2 provides the number $E_{n,k}$ of ϵ-machines for a given number of states *n* and alphabet size *k*.

Using the data in Table 2, we again consider the ratios of $E_{n,k}/B_{n,k}^{1}$. Looking at 2-state machines with an increasing alphabet, the ratio quickly approaches 1/2, indicating that almost every accessible DFA with 2 states is a topological ϵ-machine. (Recall that half of all machines are noncanonical isomorphisms).

Although data are lacking to make a definitive conclusion, there is also a trend that the number of ϵ-machines increases more rapidly with increasing states (at large alphabet) than with increasing alphabet size. This agrees with how the number of accessible DFAs grows given these two conditions, but we need more data to be sure.

At this point, we need to address two types of overcounting that appear in Table 2. The first occurs due to multiple representations of a process using a larger alphabet. For example, all machines over l≥2 letters are also machines over *k* letters for k>l. In fact, there are kl representations for each *l*-ary machine in the *k*-ary library. One may be more interested, however, in new structural features and process characteristics that appear with a larger alphabet rather than the number of ways we can re-represent machines with smaller alphabets. As such, Table 3 provides the number $F_{n,k}$ of topological ϵ-machines that employ all *k* letters. These machines cannot be found for smaller *k* and are, thus, “new” due to the larger alphabet.

The second type of overcounting is due to symbol isomorphism. Certain processes listed in both Table 2 and Table 3 have multiple representations that are different as ϵ-machines but have the same characteristics, for example, when quantified using information-theoretic measures of complexity. The Even Process, to take one example, can be considered as having even-length blocks of 1 s, as depicted in Figure 2, or even-length blocks of 0 s. The measurable process characteristics are the same for these two processes. We include both in our list, as the numbers are of interest to those studying finite-state transducers, as one example.

We also note that Table 2 and Table 3 are incomplete. This is not a shortcoming of the algorithm, but rather a comment on the exploding number of ϵ-machines. Looking only at the binary alphabet ϵ-machines, we see that their numbers increase very rapidly.

Looking at the generation times for binary alphabet machines in Table 4, we see that the run times increase very rapidly also. Our estimate for 9-state binary machines is approximately 35 CPU days. Naturally, since they depend on current technology, the absolute times are less important than the increasing ratios of run times.

## 6. Applications

Computational mechanics considers a number of different propertie, including the entropy rate, statistical complexity, and excess entropy, to quantify a process’s ability to store and transform information [5]. Additionally, there are known bounds on a number of these quantities as well as generalizations of ϵ-machines that achieve these bounds, e.g., see the binary ϵ-machine survey in Ref. [6]. However, little is known about the nonbinary alphabet case and about other more recently introduced quantities, such as causal irreversibility and crypticity. A survey of the intrinsic Markov order and the cryptic order for 6-state ϵ-machines recently appeared in Ref. [18]. A series of sequels will provide additional surveys, all of which depend on the ϵ-machine libraries we have shown how to construct.

Beyond this kind of fundamental understanding of the space of stochastic processes and the genericity of properties, ϵ-machine enumeration has a range of practical applications. One often needs to statistically sample representations of finite-memory stochastic processes, and a library of ϵ-machines forms the basis of such sampling schemes. In the computational mechanics analysis of spatiotemporal patterns in spatial dynamical systems, ϵ-machines play the role of representing spacetime shift-invariant sets of configurations. The library can then be used in computer-aided proofs of the domains, particles, and particle interactions that are often emergent in such systems. Finally, in Bayesian statistical inference from finite data, priors over the space of ϵ-machines are updated based on the evidence the data provides. Applications along these lines will appear elsewhere.

## 7. Conclusions

Beginning with an algorithm for enumerating and generating accessible DFAs, we showed how to enumerate all topological ϵ-machines based on the fact that they are strongly connected and minimal DFAs, discounting for isomorphic redundancies along the way.

There are a number of open problems and extensions to the algorithm and enumeration procedure to consider. Ideally, we would like to modify this algorithm or create an altogether new one that directly generates topological ϵ-machines without having to generate a larger class of objects—counted via $B_{n,k}^{1}$—that we then prune. Failing this, at least we would like to generate a smaller class of DFAs, perhaps only those that are strongly connected, so that fewer candidate DFAs need be eliminated.

We would also like to find a closed-form expression for the number of topological ϵ-machines for a given *n* and *k*. If this is not possible, we would like reasonable upper bounds on this quantity (better than $B_{n,k}$) and, perhaps, asymptotic estimates of the number of accessible DFAs that are actually topological ϵ-machines. Along these lines, we conjecture that for fixed *k*, $\lim_{n\to \infty }E_{n,k}/B_{n,k}^{1}=0$ and, for fixed *n*, $\lim_{k\to \infty }E_{n,k}/B_{n,k}^{1}=1/n$.

## Figures and Tables

**Table 1:** The number $E_{n,2}$ of binary-alphabet topological ϵ-machines as a function of the number of states (*n*) and edges (*k*). The number $B_{n,2}^{1}$ of accessible binary DFAs is listed for comparison.

States	Edges	$E_{n,2}$	$B_{n,2}^{1}$
1		3	
	1	2	
	2	1	
2		7	45
	2	1	
	3	6	
3		78	816
	3	2	
	4	22	
	5	54	
4		1388	20,225
	4	3	
	5	68	
	6	403	
	7	914	
5		35,186	632,700
	5	6	
	6	192	
	7	2228	
	8	10,886	
	9	21,874	
6		1,132,613	23,836,540
	6	9	
	7	512	
	8	9721	
	9	85,974	
	10	360,071	
	11	676,326	
7		43,997,426	1,048,592,640
	7	18	
	8	1312	
	9	37,736	
	10	526,760	
	11	3,809,428	
	12	14,229,762	
	13	25,392,410	
8		1,993,473,480	52,696,514,169
	8	30	
	9	3264	
	10	133,218	
	11	2,729,336	
	12	30,477,505	
	13	190,505,028	
	14	651,856,885	
	15	1,117,768,214	

**Table 2:** The number $E_{n,k}$ of topological ϵ-machines as a function of number of states *n* and alphabet size *k*.

n∖k	2	3	4	5	6
1	3	7	15	31	63
2	7	141	1873	20,925	213,997
3	78	15,598	1,658,606	136,146,590	
4	1388	3,625,638			
5	35,186				
6	1,132,613				
7	43,997,426				
8	1,993,473,480				

**Table 3:** The number $F_{n,k}$ of full-alphabet topological ϵ-machines as a function of number of states *n* and alphabet size *k*.

n∖k	2	3	4	5	6
1	1	1	1	1	1
2	7	120	1351	12,900	113,827
3	78	15,364	1,596,682	128,008,760	
4	1388	3,621,474			
5	35,186				
6	1,132,613				
7	43,997,426				
8	1,993,473,480				

**Table 4:** Average run times (2.4 GHz Intel Core 2 Duo CPU) to generate all binary alphabet topological ϵ-machines as a function of the number *n* of states.

*n*	Time (s)
3	$1.00\times 10^{-2}$
4	$1.30\times 10^{-2}$
5	$2.75\times 10^{-1}$
6	$1.39\times 10^{1}$
7	$7.80\times 10^{2}$
8	$4.94\times 10^{4}$

## Data Availability

Data is contained within the article.

## Appendix A. String-Index Mapping

Let *S* be some DFA string representation, and let *f* be the flag corresponding to *S*. Then, we have $B_{n,k}^{1}\left(s\right)=n_{f}+n_{r}$, where:

(A1) $$\begin{array}{cc} \;\;n_{f} & =\sum_{j=1}^{n-1}\left[\prod_{m=0}^{j-1}{\left(m+2\right)}^{f_{m+1}-f_{m}-1}\right.\\ \; & \;\;\;\;\;\;\times \left.\sum_{l=f_{j}+1}^{jk-1}\left({\left(j+1\right)}^{l-f_{j}}N_{j,l}^{1}\right)\right] \end{array}$$

(A2) $$\begin{array}{cc} n_{r} & =\sum_{j=1}^{n-1}\left[\sum_{l=f_{j}+1}^{f_{j+1}-1}s_{l}{\left(j+2\right)}^{f_{j+1}-1-l}\right.\\ \; & \;\;\;\;\times \left.\left(\prod_{m=j+1}^{n-1}{\left(m+2\right)}^{f_{m+1}-f_{m}-1}\right)\right]. \end{array}$$

Equation (A1) calculates the first index that uses the given flag, and Equation (A2) calculates the index of the string *S* among those DFAs with the given flag.

Equation (A1) refers to the number $N_{j,l}^{1}$ of accessible DFAs whose string representation has the first occurrence of symbol *j* occur in position *l*. It can be defined by a recursive formula and its values stored in a table for efficient access. For completeness, we provide the formulas here, but for more detail we direct the reader to Refs. [8,9]:

$$\begin{array}{cc} N_{n-1,j}^{1} & ={\left(n+1\right)}^{nk-1-j}\;,j\in \left[n-2,\left(n-1\right)k-1\right]\\ N_{m,mk-1}^{1} & =\sum_{i=0}^{k-1}{\left(m+2\right)}^{i}N_{m+1,mk+i}^{1}\;,m\in \left[1,n-2\right]\\ N_{m,j}^{1} & =\left(m+2\right)N_{m,j+1}^{1}+N_{m+1,j+1}^{1}\;,\\ \; & \;\;\;m\in [1,n-2],j\in [m-1,mk-2]\;. \end{array}$$
